# Amphoteric dissolution of two-dimensional polytriazine imide carbon nitrides in water

**DOI:** 10.1098/rsta.2022.0339

**Published:** 2023-10-30

**Authors:** Karolina Lisowska, Will Purser, Fuqiang Chang, Theo M. Suter, Thomas S. Miller, Andrea Sella, Christopher A. Howard, Paul F. McMillan, Furio Corà, Adam J. Clancy

**Affiliations:** ^1^ Department of Chemistry, University College London,London WC1E 0AJ, UK; ^2^ Department of Chemical Engineering, University College London, London WC1E 7JE, UK; ^3^ Department Physics and Astronomy, University College London, London WC1E 6BT, UK

**Keywords:** carbon nitride, polytriazine imide, PTI, green chemistry

## Abstract

Crystalline two-dimensional carbon nitrides with polytriazine imide (PTI) structure are shown to act amphoterically, buffering both HCl and NaOH aqueous solutions, resulting in charged PTI layers that dissolve spontaneously in their aqueous media, particularly for the alkaline solutions. This provides a low energy, green route to their scalable solution processing. Protonation in acid is shown to occur at pyridinic nitrogens, stabilized by adjacent triazines, whereas deprotonation in base occurs primarily at basal plane NH bridges, although NH_2_ edge deprotonation is competitive. We conclude that mildly acidic or basic pHs are necessary to provide sufficient net charge on the nanosheets to promote dissolution, while avoiding high ion concentrations which screen the repulsion of like-charged PTI sheets in solution.

This article is part of the theme issue 'Exploring the length scales, timescales and chemistry of challenging materials (Part 2)'.

## 1. Introduction

Graphitic carbon nitrides (gCNs) are a class of metal-free semiconductors with bandgaps [1,2] ranging from 1.73 eV to 2.88 eV (most commonly approx. 2.7 eV) that are the subject of intense research owing to their low cost, relatively simple and scalable synthesis, thermal and chemical stability and high surface area. Their specific characteristics can be determined by a combination of molecular precursor(s), synthetic conditions, potential doping and post-processing. The optical and electronic properties make gCNs promising candidates and alternatives for a wide range of energy and environmental applications including photocatalysts [3], electrolysers [4], photovoltaic cells [5], solar batteries [6] and light emitting diodes [7]. They have also been screened for applications in anti-microbial systems [8], heavy metal sensors [9], and water purification [10].

Most commonly, the literature describes gCNs prepared by the thermolysis of nitrogen-rich organic precursors; such processes yield more or less extensively crosslinked but amorphous heptazine polymers with partial alignment into local two-dimensional domains [11]. It should be noted that the term g-C_3_N_4_ is occasionally erroneously used to describe these materials, alluding to the fully condensed, hydrogen-free theoretical structure of triazines/heptazines linked through tertiary amine bridges of stoichiometry C_3_N_4_, which has proven challenging to reliably synthesize [11–13].

**Figure 1.**
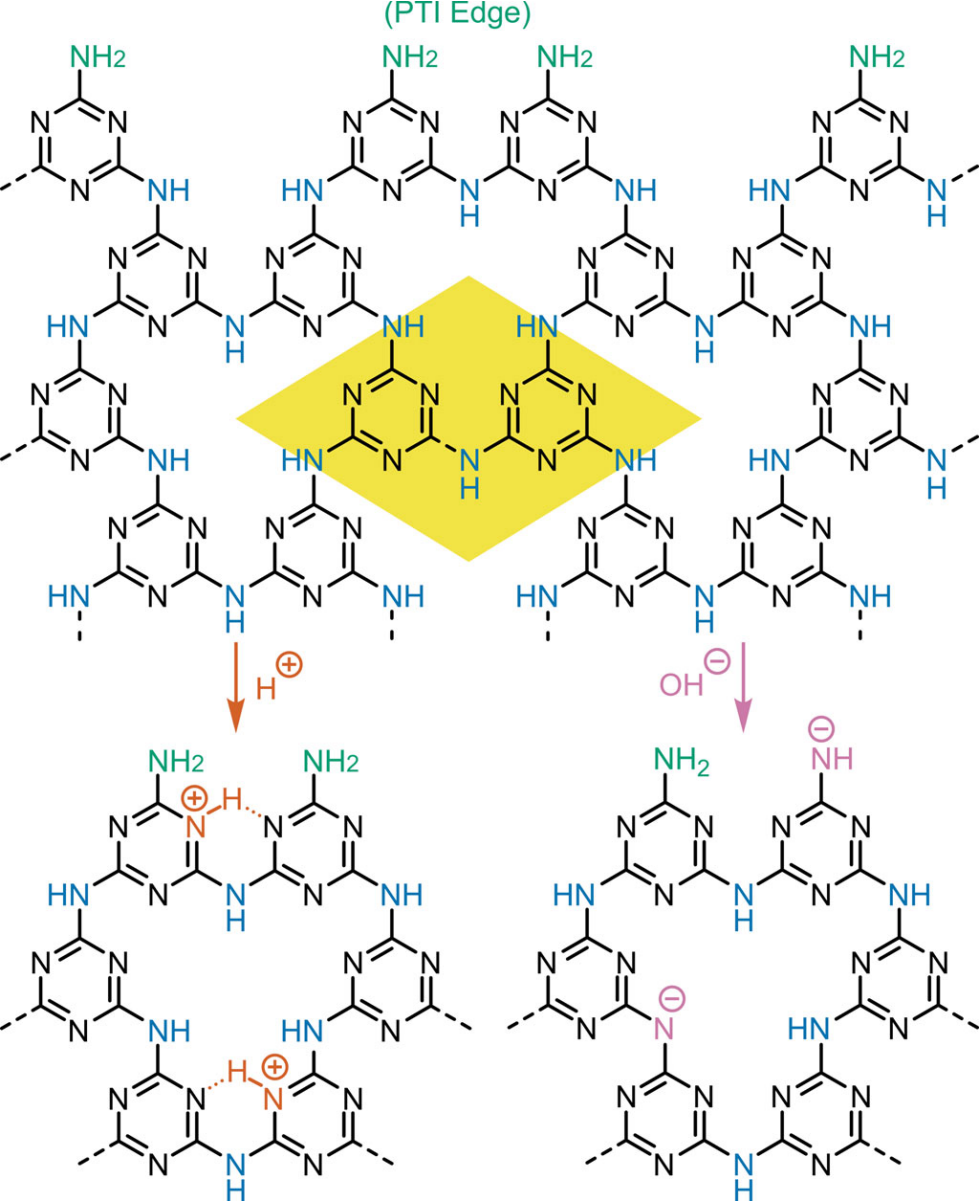
Structure of PTI sheet before (top) and after acid (left) and base (right) treatments. Colour highlighting for edge sites (green), imide bridges (blue), protonated sites (red) and deprotonated sites (purple). Unit cell highlighted in yellow.

By contrast, highly crystalline two-dimensional carbon nitrides are now well-established. Two-dimensional porous sheets based on triazines linked with NH bridges forming hexagonal C_6_N_9_H_3_ unit cells, are referred to as polytriazine imide (PTI, figure 1). More recently, analogous fully crystalline sheets of NH bridged heptazines (polyheptazine imide, PHI, C_12_N_17_H_3_) containing larger intralayer pores have also been synthesized [14,15]. In both structures the covalently bonded layers stack in an ordered fashion determined by the nature of, typically, alkali metal halide intercalants, with van der Waals interactions holding one layer to the next. The two structures differ not only in the structural units but also in the size of the intralayer pores which are substantially larger for PHI than for PTI.

While PTI was originally synthesized on very small scales through high pressure experiments [16], it can now be routinely obtained in larger quantities through condensation of nitrogen-rich precursors in eutectic salt baths [17,18] (LiX/MX, X = Cl/Br, M = K/Na for PTI). Prepared through this route, the as-synthesized material consists of prisms of stacked hexagonal sheets containing intercalating LiX salt—sodium/potassium intercalation is unfavourable [19]—as well as a small lithium excess substituting in-plane hydrogens (LiX · C_6_N_9_H_(3-a)_Li_a_, termed LiX · PTI). Common precursor organics include dicyandiamide and melamine, although as the synthesis involves a balance of condensation and depolymerization, the triazine-based PTI may be formed from heptazine-based precursors such as melon [20]. The polymerization occurs at approximately 550°C with reaction at this temperature leading to low defect-density PTI. While the use of 600°C is common (including in this work), the proximity to the carbonization temperature may lead to partial damage to the structure, most clearly seen as darkening of the resultant more nitrogen-poor product, and a broadening of the pXRD peaks [21]. The salt mixture selected can have significant effects on the properties of the resultant carbon nitride. While LiCl/KCl is the most common approach, the use of eutectic LiCl/NaCl can lower defect formation by more closely matching the melting point to the polymerization initiation temperature to prevent the aforementioned carbonization [22]. Contrastingly, use of mixed iodides (LiI/KI) in lieu of X = Cl/Br eutectic compositions prevents formation of PTI altogether, with the larger anions disrupting the condensation to form one-dimensional melon chains stacked in aligned sheet-like arrays [23].

The lithium halide in LiX · PTI may be removed by continuous extraction with water, yielding water-intercalated material, H_2_O·PTI. Interestingly, the water molecules which reside within the PTI pores are mobile, able to move through the material normal to the layers, travelling in single file through aligned pores [24]. The intercalated water may be removed by heating under vacuum to give pure intercalant-free C_6_N_9_H_3_ (IF-PTI) which can be reintercalated with new species such as hydrochloric acid (HCl · PTI) [25]. The IF-PTI material shows a lower degree of symmetry of the CN stacks, coupled to a change in stacking ordering, underlining the importance of the intercalants in aligning the sheets. More recently, ion-exchange of LiCl·PTI with concentrated HCl forms HCl · PTI which may be deintercalated in the solid phase by heating, leaving IF-PTI with maintained aligned-pore stacking order [26].

The intrinsic structure of PTI provides several sites suitable for Brønsted acid–base reactions: the bridging NH imide groups in PTI may be deprotonated, and protonation may, in theory, occur at these bridges or on the triazine nitrogen atoms in the basal plane (figure 1). Additionally, the often-small lateral dimensions [10,27] of eutectic salt bath-synthesized PTI (30–50 nm) leads to a relatively high proportion of edge sites containing primary amines, NH bridges and triazines for (de)protonation. The deprotonated PTI structure manifests locally in the lithium in-plane substitutions of as-synthesized LiX · PTI. The aqueous deintercalation procedure of LiX · PTI is known to lead to the substitution of lithiated imide bridges to form NH bridges through water deprotonation, forming LiOH solution in the process. This exchange was first inferred in the aqueous sonication of LiCl·PTI by Schwinghammer *et al.* [28], which led to alkali dispersions of few-layered PTI. Conversely, the structure of protonated PTI is present in the original PTI synthesis [16] through high-pressure solid-state reaction of melamine and cyanuric chloride, which led to HCl-intercalated PTI (C_6_N_9_H_4_Cl; HCl · PTI), which may also be synthesized through HCl reintercalation or ion exchange of IF-PTI and LiCl·PTI, respectively [25,26]. The protonated monolayer HCl·PTI analogue has been recently reported by Bojdys [21] through dilution in HCl solution of probe sonicated and centrifuged aqueous LiBr·PTI suspensions. The reaction led to a red-shift of the existing backbone organic peak (200–300 nm) in the UV-vis absorption spectra, in addition to the emergence of a new peak at 260 nm, assigned to symmetry-breaking from protonation of the triazine rings.

The large total inter-layer van der Waals forces in layered materials typically hinder their exfoliation to individual or few-layer sheets in a liquid phase [29]. The most common exfoliation approach is to separate the layers with shear force (e.g. from sonication) while kinetically trapping the layers to prevent reagglomeration with a surfactant solution or amidic solvents. This energy intensive and difficult-to-scale process damages the two-dimensional framework and leads to only a fraction of monolayer species in dispersion, while leaving contaminating, difficult to remove (and often environmentally harmful) species coating the material. By contrast, certain two-dimensional nanomaterials may spontaneously dissolve through *chimie douce* methods, using a chemical driving force to exfoliate individual two-dimensional layers which enter the liquid phase at room temperature, leading to an intrinsically scalable, low energy processing route towards high quality two-dimensional nanomaterials. Spontaneous dissolution is most commonly performed through reduction with group 1 metals to give salts containing anionic two-dimensional layers which dissolve in polar aprotic organic solvents, driven by solvation of the anions and counter-cations [30,31]. A less common alternative is to imbue cationic charge through acidification, often requiring the use of superacids such as chlorosulfonic acid due to the non-basic nature of many two-dimensional nanomaterials [32]. Amorphous heptazine based gCNs have been dissolved in highly acidic media, providing access to high concentrations [33] but is often associated with partial depolymerization of the amorphous network [34].

While PTI may also be subjected to the reductive redox strategy [35], unusually it has been shown to spontaneously dissolve without redox reactions in certain solvents such as dimethylsulfoxide [27] and dimethylacetamide [10]. The dissolution of neutral layered materials in non-reactive media is rare, but has been reported for other systems including two-dimensional borophene oxide [36] and layered zinc hydroxide [37]. Upon spontaneous dissolution of bulk PTI, the dissolved material may contain a mixture of monolayer and multiply stacked PTI sheets, with a greater fraction of stacked material found in higher concentration solutions [19]. Similarly, IF-PTI has a greater propensity to slightly restack in solution versus LiX · PTI, attributed to lithium ions in the latter adsorbing to the PTI to give a mild surface charge to limit restacking. While promising, this approach still requires large quantities (greater than 1 L g_(PTI)_^−1^) of environmentally hazardous solvent to dissolve the PTI sheets.

Here, the intrinsic chemistry of PTI is explored and exploited to form aqueous solutions of PTI using common acids and bases, to provide a scalable, low-cost, environmentally friendly route to creating solutions of highly crystalline two-dimensional carbon nitrides.

## 2. Methods

### (a) Materials

Dicyandiamide (99%), LiBr (anhydrous, ≥99%) and KBr (anhydrous, ≥99%) were purchased from Sigma Aldrich. NaOH (99.9%), HCl (37%) and water (HPLC grade) were purchased from VWR Limited.

### (b) PTI synthesis

LiBr·PTI was synthesized as performed in our previous work [19] adapting the synthesis of Bojdys *et al*. [17] In brief, dicyandiamide (2.0 g) was ground with dried KBr (4.8 g) and LiBr (5.2 g), heated to 400°C under N_2_ for 1 h, before sealing in a quartz ampule under vacuum and heating to 600°C for 16 h, washing with water to remove excess salt, to give a light brown powder of LiBr·PTI. The coloration is indicative of slight carbonization [21], common to PTI samples synthesized at 600°C. Subsequent deintercalation was performed to give H_2_O·PTI via Soxhlet extraction with water for 2 days and dried in a Petri dish in air to give a lighter brown powder. Further characterization data provided in electronic supplementary material, electronic supplementary material, figure S1.

### (c) pH measurement

Solution pH was measured using a SciQuip Economic Benchtop pH Meter, calibrated using commercial buffer solutions of pH 4.01, 7.00 and 10.01, in automatic compensation mode. Stock solutions of desired pH were created from adding water to approximately 2 M HCl or NaOH solutions to give solution within ±0.02 of the desired pH. Solutions were stored sealed and pH was measured immediately prior to use.

### (d) PTI dissolution

8.0 mg of either LiBr·PTI or H_2_O·PTI was placed evenly at the bottom of a 30 ml glass screw-top vial. A pre-prepared solution of desired pH (8.0 ml) was added over the PTI, the vial was flushed with N_2_, and the vial caps sealed with PTFE tape, and the mixtures gently stirred overnight. The solutions were centrifuged gently (100 rpm, 15 min, Hettich EBA 20 centrifuge) to condense the PTI powder into a pellet to allow manual collection of the solution (approx. 7 ml) without agitating the solid PTI.

### (e) UV-vis spectroscopy

UV-Vis spectra were recorded on a Shimadzu UV-2401 in a Helma A UV quartz micro-rectangular cuvette (pathlength 10 mm), subtracting an air background.

### (f) Thermogravimetric analysis

TGA were recorded on a Perkin Elmer Pyris 1 TGA, with approximately 5 mg of material under 60 ml min^−1^ N_2_ flow, holding at room temperature for 5 min to outgas the sample before heating at 10°C min^−1^ to 800°C.

### (g) FTIR

Fourier transform infrared spectra were recorded on a Bruker Alpha Fourier transform infrared spectroscopy instrument between 400 and 4000 cm^−1^ for 16 averaged scans at 0.25 cm^−1^ resolution with autosubtracted background.

### (h) Powder X-ray diffraction

XRD was performed on a Malvern PANalytical X'pert Pro, using a Cu K*α* source between 0 and 70° 2*θ* in 0.05° increments.

### (i) Photoluminescence

Photoluminescence (PL) spectra were recorded on a Horiba Scientific Flourolog in three-dimensional acquisition mode, using a 10 × 4 mm pathlength optical glass cuvette. Measurements were taken using excitations between 250–450 nm in 5-nm increments, with emission recorded between 300–500 nm in 1 nm increments. Data presented are detector intensity (in CPS) corrected by subtraction for background light and normalized by the light intensity (as measured in μA). Data presented by normalizing to the most intense signal, not including elastic scattered peaks. Full scaling values provided in, electronic supplementary material, table S1.

### (j) Zeta potential

Dynamic light scattering measurements were performed using a Malvern Zetasizer Ultra. Diluted aqueous dispersions were ultrasonicated for 3 min before measured at 25°C. An equilibration time of 2 min was applied before starting each experiment to ensure that the dispersions were at the set temperature. The measurements consisted of six runs of 15 individual data acquisitions. Data were normalized by intensity with runs averaged using the *Average Multiple Curves* function in OriginPro.

### (k) Transmission electron microscopy

Transmission electron microscopy measurements were performed on a JOEL 2100 in diffraction mode with an accelerating voltage of 200 keV. To prepare samples, a carbon-film copper grid (Agar Scientific) was placed on top of a Whatman cellulose filter paper, and approximately 30 µL of as-dissolved solution was dropped on top and left to dry at room temperature overnight.

### (l) Density functional theory

Density functional theory (DFT) calculations were implemented with the code CRYSTAL17, using the hybrid exchange functional B3LYP (Becke, 3-parameter, Lee–Yang–Parr) with D3 corrections and periodic boundary conditions in three dimensions (*bulk*), two dimensions (*surface* and *monolayer*), one dimension (*ribbon*) [38–40]. A semi-empirical geometric counterpoise correction has been used to remove artificial overbinding effects arising from the Basis Set Superposition Error [41]. The all-electron Gaussian basis sets of all atoms were obtained from the CRYSTAL online database, including hydrogen [42] (H_3-1p1G_ gatti_1994), carbon [42] (C_6-31d1G_gatti_1994), nitrogen [42] (N_6-31d1G_gatti_1994), oxygen [42] (O_6-31d1_gatti_1994), chlorine [43] (Cl_86-311G_apra_1993) and sodium [44] (Na_8-511G_dovesi_1991). Full geometry optimizations have been performed for all systems using the default converge criteria of CRYSTAL17. Atom colours are represented using a scale optimized for colour blind individuals proposed by Bang Wong [45], selecting options most similar to the classic CPK colouring convention.

## 3. Results and discussion

To experimentally probe the PTI acid–base chemistry, LiBr · PTI was synthesized from dicyandiamide in LiBr/KBr using literature methods, and H_2_O · PTI was synthesized through Soxhlet extraction of LiBr · PTI and dried at room temperature in air. Since water spontaneously intercalates IF-PTI when added to an aqueous solution, here H_2_O·PTI was used as the salt-free PTI material to avoid unnecessary drying, in contrast to our previous experiments dissolving IF-PTI in anhydrous solvents [19]. A series of aqueous solutions from pH 0–14 were prepared from deionized water using HCl and NaOH; each solution (8 ml) was added to each PTI type (8 mg), and the vials were flushed with nitrogen, sealed with PTFE tape to prevent atmospheric CO_2_ ingress (which might lower the measured final pH through formation of carbonic acid) and left overnight.

**Figure 2.**
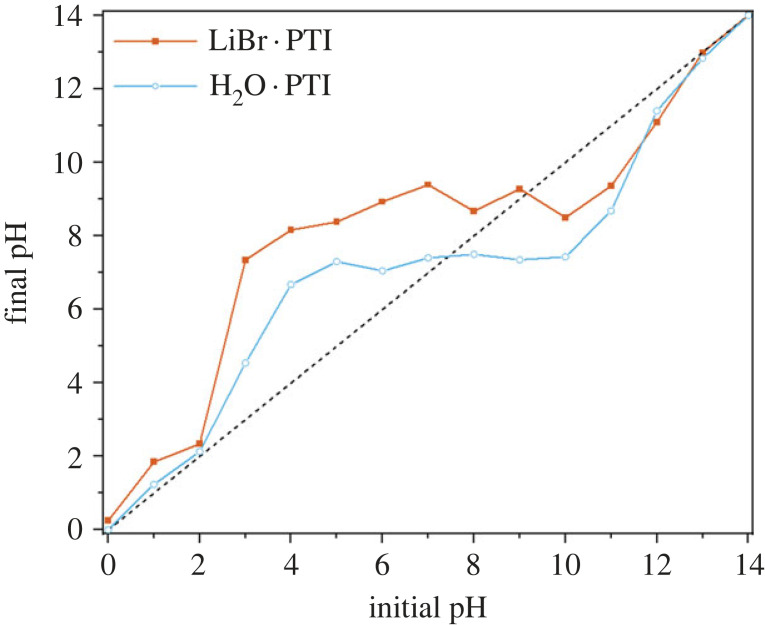
Initial pH of aqueous solution versus final pH of solution 2 days after adding H_2_O · PTI (blue open circles) or LiBr · PTI (red squares) powder. Dashed line of unchanged pH is provided to highlight deviations.

The pH of the solutions at equilibrium was then measured and plotted against the initial pH (figure 2). The PTI demonstrated amphoteric behaviour, with the H_2_O · PTI buffering the pH at approximately 7.2, confirming the presence of both acid and base reactivity of the PTI (N.B. the locus of these reactive sites and resultant structures is discussed extensively below). Starting from the solutions with lower pH values (2–7), the PTI can be seen to decrease the pH through removal of H^+^ from solution, therefore acting as a base, while high initial pH values (8–13) led to deprotonation of the PTI, decreasing the OH^−^ concentration in solution. A similar amphoteric behaviour was seen for LiBr · PTI, although the buffering occurred at approximately pH 9. The offset may be partially attributable to a depletion of H^+^ from reaction with the in-plane lithium substitution (equivalent to the conjugate base from LiOH PTI deprotonation).

**Figure 3.**
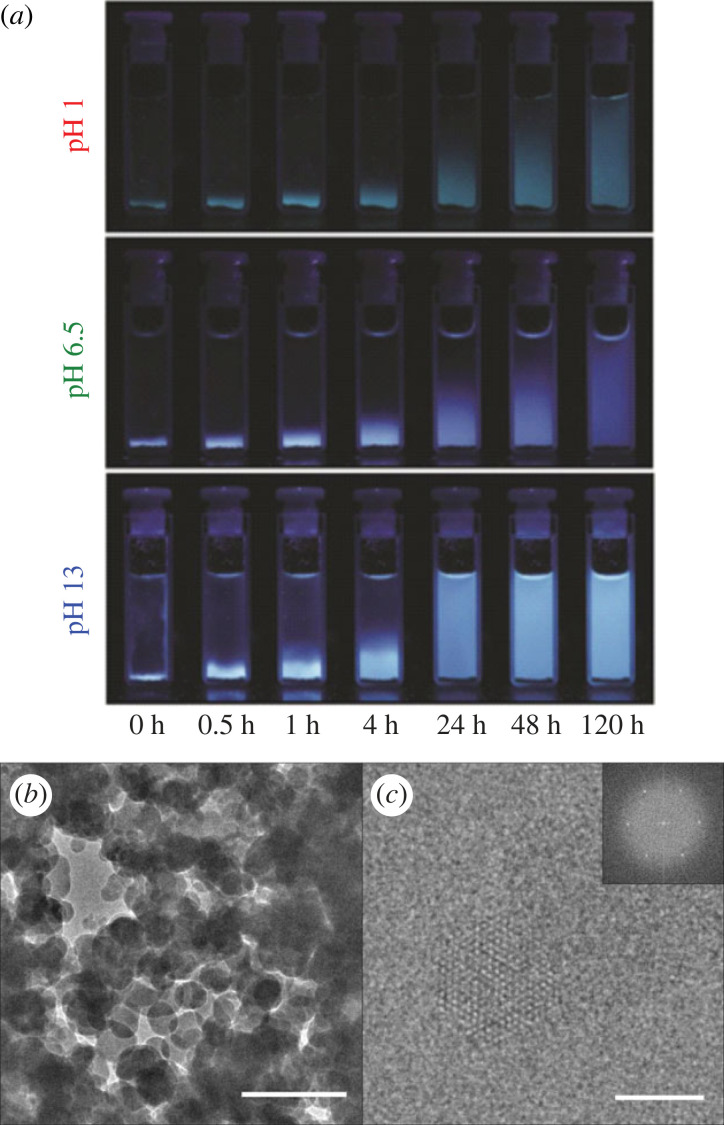
(*a*) Digital photographs under long-wavelength (less than 365 nm) UV illumination of LiBr·PTI submerged in aqueous HCl solution (pH 1, top), deionized water (pH 6.5, middle) and NaOH solution (pH 13, bottom) left undisturbed at room temperature over 5 days. (*b,c*) Transmission electron micrographs of LiBr · PTI dissolved at pH 7. (*a*) Scale bar 200 nm. (*b*) Scale bar 100 nm with fast Fourier transform inset.

The solutions became yellow overnight, indicating dissolution of PTI flakes, with a front of dissolving PTI rising from the powder seen under UV light (figure 3), although a significant fraction of the PTI remained solid in all cases, as occurs in DMF [27] and DMSO [19]. The solutions were analysed by UV-visible spectroscopy showing several distinct peaks, in addition to a broad exponential background covering the entire UV-visible range attributed to Tyndall scatter, indicative of particles of PTI dispersed in the liquid [31]. As no agitation was necessary for the dissolution, it may be inferred that the process is thermodynamically favourable, in contrast to typical shear-based nanomaterial solution processing (e.g. sonication) which may lead to metastable suspensions: here we use the term ‘solvation’ to distinguish the thermodynamic products from kinetically stabilized dispersion/suspension.

**Figure 4.**
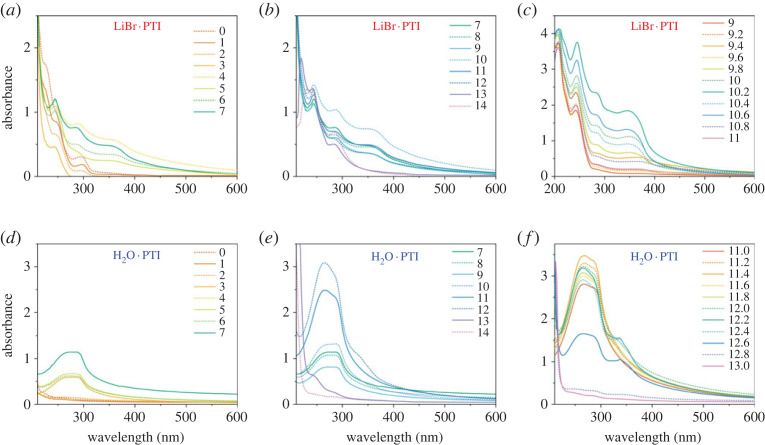
UV-vis spectra of (*a–c*) LiBr · PTI and (d-f) H_2_O · PTI dissolved in HCl/NaOH solutions of varying pH. For clarity, acidic and basic solutions in 1 pH increments are presented separately (*a,d*) and (*b,e*), respectively, and solutions around the maximum absorbance pH in 0.2 increments are given for (*c*) LiBr · PTI and (f) H_2_O · PTI. Intensity profiles of select peaks provided in, electronic supplementary material, figure S5.

**Figure 5.**
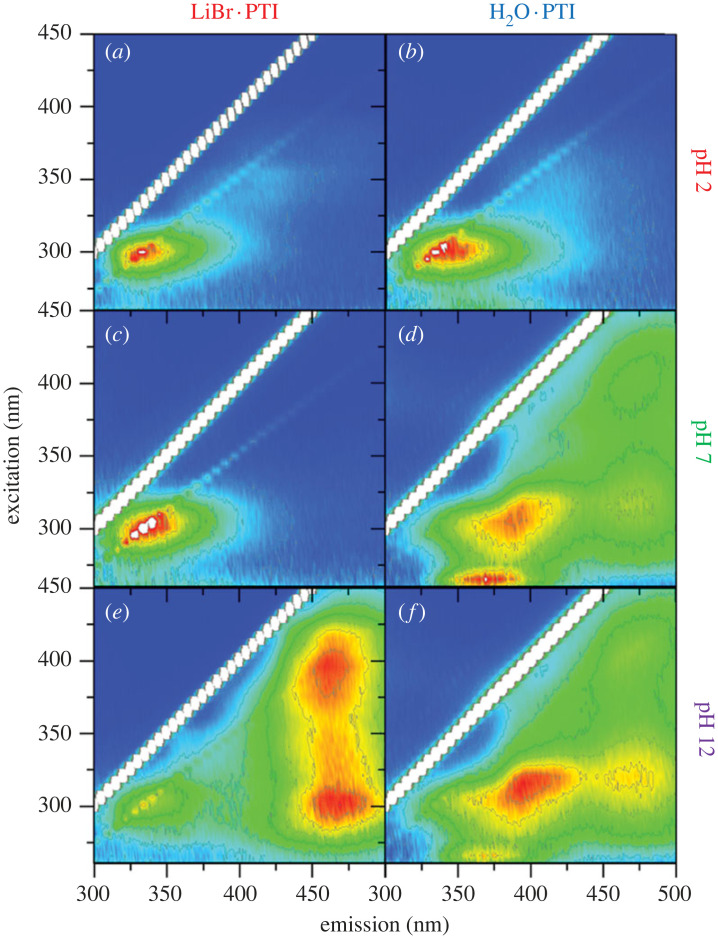
Photoluminescence maps of (*a,c,e*) LiBr · PTI and (*b,d,f*) H_2_O · PTI solutions in aqueous solutions of initial acidity of (*a,b*) pH 2, (*c,d*) pH 7 and (*e,f*) pH 12. Signal intensity normalized to highest non-elastic signal (electronic supplementary material, table S1) on a blue-red scale with contour lines used to bin data at 1/8 intensity intervals.

The UV-vis spectra (figure 4) and PL maps (figure 5) provide additional insight into the character of the PTI solutions. The UV-vis spectra of LiBr · PTI consists of several distinct peaks, which are in broad agreement with previously reported data [21,25,27]. The peak at approximately 290 nm is attributed to the CN backbone; the broader peak centred around 370 nm has been previously attributed to alkali metal-substituted NH bridges, while the peak at 260 nm has been attributed to triazine protonation. The relative quantity of dissolved PTI may be inferred from the UV-vis spectra, although accurate quantification is complicated by potential solvatochromism and multiple overlapping peaks arising from interdependent phenomena and local structures which change as a function of pH and PTI starting material. Filtration was attempted, but was unreliable due to the small total masses involved, although DMSO solutions of comparable appearance to the most concentrated solutions here have previously been quantified through filtration [5] as 60 µg ml^−1^.

The dissolution of LiBr · PTI can be seen to be negligible in highly acidic conditions (pH ≤ 3) which have acidic final pHs (figure 2), with no peak seen in the 350 nm region, with the approximately 290 nm peak present throughout, red-shifting in more acidic media as proposed by Bojdys and colleagues for acidified sonicated PTI dispersions [21]. Notably, recent developments involving acid-PTI interactions has involved pH ≤ 0 aqueous solutions, where no spontaneous dissolution is expected [21,26]. At these low pH values, the surface charge on the PTI sheets is shown to be near neutral (electronic supplementary material, figure S3). The PL highlights the presence of PTI in solution at pH 2 (figure 5*a*) with an excitation (*λ*_ex_) at 300 nm, with linked emission (*λ*_em_) at 340 nm, near matching the 294 nm UV absorption seen for LiBr·PTI in pH 2 HCl. The quantity of liquid-phase PTI dramatically increases at pH 4, linked to the significant change in pH into the buffering region indicating that in these conditions the LiBr·PTI behaves as a base, forming more positive PTI sheets, both from removal of Li-substituted bridge sites and protonation of the PTI sheet (which is the only effect rationalizing the buffering of H_2_O · PTI in acidic media, figure 2). Notably, in the buffering region, the charge on the PTI sheets is negative (more-so for LiBr·PTI than IF-PTI at any given pH, attributed to the higher fraction of Li-substituted bridges, electronic supplementary material, figure S3). The presence of the 350 nm peak in the UV-vis spectrum indicates that some imide bridges remain deprotonated (figure 4*a*).

Moving further towards neutrality, as pH increases towards 6–9 a smaller positive charge is expected from lessened PTI protonation in milder conditions, which is supported by the lessened red-shift of the approximately 290 nm peak, down to a minimum at pH 7. Mildly basic initial solutions (pH 10–12) see a subsequent increase in dissolution linked to PTI deprotonation, forming anionic C_6_N_9_H_(3-y)_^y−^ sheets, as seen through the solution pH decrease. The increased PTI negative charge within the buffering region is accompanied by significant increases in absorption in the UV-vis spectra. These high levels are coupled to the emergence of two PL signals with *λ*_em_ 480 nm. This first peak with *λ*_ex_ 400 nm has been used previously to assign approximately 370 nm absorption to Li-substituted bridges (although the full excitation range was not mapped and the primary counterion here is Na^+^). The second peak at *λ*_ex_/*λ*_em_ 300/480 nm is associated with the CN framework excitation, but is absent in previous measurements on pre-exfoliated PTI monolayers [21] We preliminarily assign this peak to multiply stacked PTI in solution, which reflects comparable effects measured in stacked solid PTI due to lessened confinement in the interlayer plane, modelled using a particle-in-a-box quantum well model [27].

Maximum LiBr·PTI absorption occurs at pH 10.2 (figure 4*c*), which coincides with the greatest relative intensity of the approximately 350 nm peak of deprotonated bridges, indicating maximizing negative charge on the sheets leads to the aqueous dissolution. Increasingly alkaline solutions (pH ≥ 10.4) show lower total absorption as the pH is raised outside the buffering window, attributed to typical salting out of the solutions from the increased ionic strength of the solution screening repulsive-like charges [46,47]. This salting out can be seen for the highly acidic systems, where there is significantly lower levels of PTI in the aqueous phase as measured by UV-vis spectroscopy in these high ionic strength solutions.

The behaviour of H_2_O · PTI is subtly different from the LiBr · PTI. Most notably, while the UV-Vis spectra also demonstrate the main peaks observed for the intercalated material, the peaks are notably broader in all cases, perhaps due to the more disordered nature of the post-deintercalation PTI [25]. The buffering of PTI in acidic media (figure 2) confirms that the PTI may act as a base, becoming positively charged at highly acidic pHs (electronic supplementary material, figure S3), with UV-vis absorption decreasing with increasing acidity (figure 4*d*) as seen for LiBr·PTI. In basic media, the PTI dissolves more strongly, with a maximum seen at pH 11.4, above which increasing basicity decreases absorption in the UV-vis spectra. The approximately 350 nm peak is seen at notably shorter wavelengths and is present in a narrow pH window (11.8–12.6, figure 4*f*). Assuming this excitation requires delocalized π/π* systems to stabilize the anionic charge, this effect is consistent with lower crystallinity versus LiBr·PTI, with lessened delocalization from less-planar PTI causing greater confinement and associated higher absorption energies, although the possibility of less-stacked aggregates cannot be discounted. The PL behaviour is likewise broadly similar to the LiBr·PTI with broader signals (figure 5), although a clear difference is seen at pH 7, where the *λ*_ex_/*λ*_em_ peaks of 300/470 nm and 400/470 nm are absent, attributed to the neutral final pH of the solution whereas LiBr·PTI in initial pH 7 solution was mildly basic (figure 2). At pH 12, these PL peaks are present for H_2_O · PTI, indicating the presence of deprotonated bridges, and stacked species in solution, respectively.

The pH dependent stability of the solutions was further demonstrated through deliberate destabilization by altering the pH. A solution of H_2_O·PTI dissolved at pH 11.4 was decanted and diluted with equivalent volume of 12 M HCl solution and allowed to settle over 1 h before decanting the top fraction. While simple dilution effects would lead to UV-vis absorption halving, here the absorption of the low wavelength peaks decreased by over four times (electronic supplementary material, figure S6) indicating precipitation of the PTI from solution.

IR spectroscopy of the H_2_O·PTI (electronic supplementary material, figure S4) provided insight into the chemical behaviour of the PTI before and after treatment with acid (pH 2) and base (pH 12). Acidification leads to formation of a new broad signal around 2925 cm^−1^, indicative of the formation of additional, weaker N-H bonds, consistent with acidification. Concurrently, the peak maxima at 1265 and 1181 cm^−1^ broaden and downshift to 1253 and 1177 cm^−1^, respectively, implying weakening of the C-N bond of the imide bridge. A new, weak, unassigned mode also arises at 894 cm^−1^. Treatment with base has a less dramatic effect on the IR spectrum than acidification. Surprisingly the N-H region is virtually unaffected, indicative of a significant fraction of the N-H bonds remaining after reaction, in spite of the more dramatic influence on the dissolution behaviour in comparison to HCl treatment. The C-N stretch 1265 cm^−1^ peak downshifts to 1259 cm^−1^ from bond weakening, and a pronounced shoulder around 1212 cm^−1^ emerges, while the peak at 1181 cm^−1^ becomes vanishingly weak. Notably, in all cases, the peaks most commonly assigned to triazine vibrations (approx. 1650 cm^−1^ C = N stretches, 800 cm^−1^ C_3_N_3_ ring bend) appear relatively unchanged.

A more detailed mechanism of acid/base reactivity of the system was explored through DFT calculations (B3LYP with D3 correction using CRYSTAL17), using four IF-PTI models. Firstly, a three-dimensional periodic PTI model is used to model bulk PTI (de)protonation (*bulk*). Secondly, a five-layer slab with periodicity in the *ab* plane is used with acid/base addition occurring at the top layer (*surface*), allowing an understanding of how reactions occur on the surface layer of a PTI stack where the sub-surface layers are unreacted. Thirdly, a PTI monolayer is used to compare acidification/basification in the absence of interactions with adjacent PTI layers (*monolayer*). Finally, a one-dimensional PTI monolayer ribbon one pore in width with -NH_2_ terminations is used to model the effects of edge sites, in contract to internal basal plane modifications (*ribbon*). The starting structure for the DFT calculations has crystalline sheets stacked in the order characteristic for the IF-PTI with triazine rings in adjacent layers slightly displaced relative to each other along the [010] direction [24]. Atoms were then allowed to fully relax and occupy their preferred minimum energy locations. To model the interactions with acid, each PTI model was relaxed with the addition of a HCl molecule with the hydrogen initially localized adjacent to a NH bridge or triazine nitrogen to undertake the reaction (C_6_N_9_H_3_)_n_ + HCl − > [C_6n_N_9n_H_3n + 1_^+^][Cl^−^], with energy of protonation (Δ*E*_prot_) defined as the difference in energy of the protonated structure (*E*_HCl·PTI_) and the sum of the energy of the neutral parent PTI structure (*E*_PTI_) and HCl molecule (*E*_HCl_) in vacuum, using equation (3.1).

3.1 $$\Delta E_{\text{Prot}}=\;E_{\text{HCl}\cdot \text{PTI}}-\;E_{\text{PTI}}-\;E_{\text{HCl}}.$$

**Figure 6.**
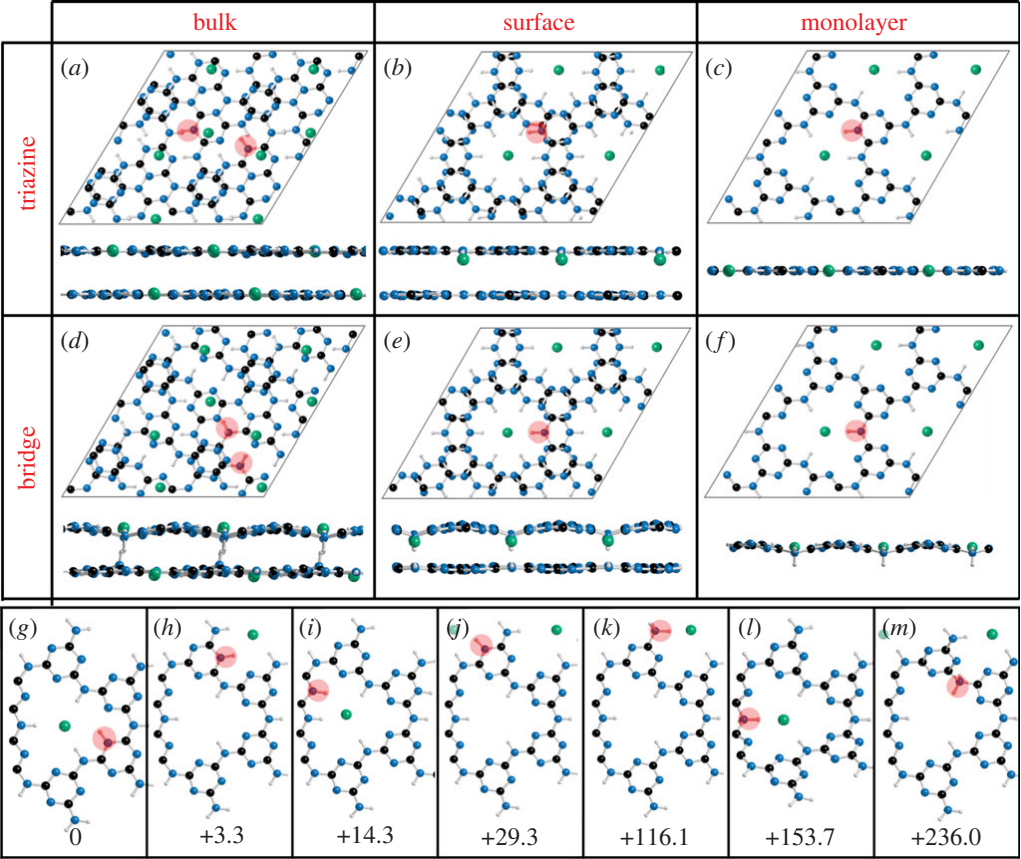
Protonated PTI structures from DFT calculations. (*a–f*) 2 × 2 × 1 supercell visualized down the a-axis (bottom) and along the c axis (top). Surface models show near-along c-axis as to align the imide bridges for clarity. Structures given for (*a–c*) triazine protonation and NH bridge protonation (*d–f*) of (*a,d*) bulk, (*b,e*) top two layers of the surface model, and (*c,f*) monolayer. (*g–m*) Ribbon model structures of protonation sites near PTI edges, arranged in order of Δ*E*_prot_ (in kJ mol^−1^) relative to the most stable. Red circles used to highlight site of protonation (N.B. only one site highlighted in b-f supercells, and two sites for bulk, with one on each layer in the unit cell). Additional surface models (directly along c-axis, all five layers down a-axis) and ribbon models visualized along a-axis provided in electronic supplementary material, figure S8.

**Figure 7.**
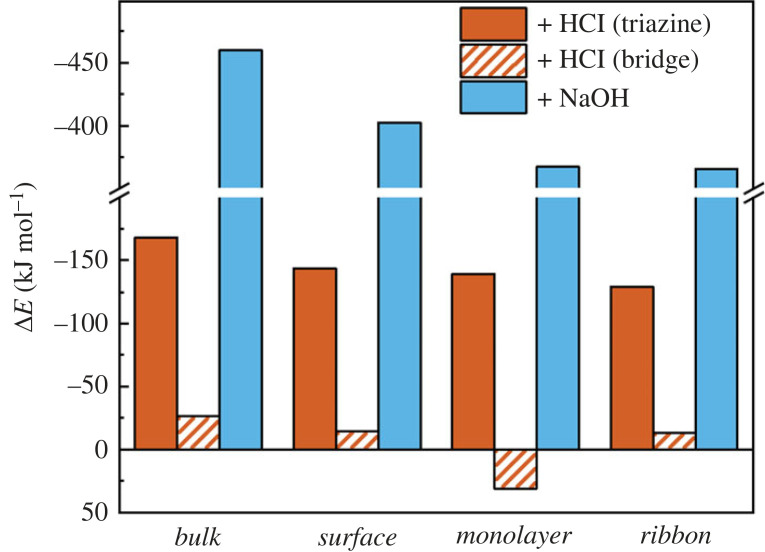
ΔEprot (red) at triazine (solid) and NH bridge (dashed) sites, and ΔEdeprot of NH bridges (blue solid) calculated from equation (3.1) and equation (3.2), respectively. Most favourable (de)protonation site value given for each model type, with full values for all models, provided in electronic supplementary material, tables S5 and S6.

The preferred site of protonation in all cases is at the triazine ring, leading to an in-plane N-H bond (≈ 1.0 Å, electronic supplementary material, table S3) with the hydrogen directed towards the lone pair of an adjacent triazine's nitrogen (≈ 2.0 Å, electronic supplementary material, table S3), lying in the plane of the PTI (figure 6*a–c*). The bond motif matches the XRD-derived structure in solid HCl·PTI [16,25] and proposed for acidified pre-exfoliated monolayers [21], while the bond lengths closely match those of the pyridine-pyridinium hydrogen bonded dimer in water [48] (1.02/1.98 Å, respectively). This hydrogen bond formation within the voids of the PTI stabilizes the protonated structure (*vide infra*) and all triazine protonation sites yield negative energies, indicating favourable protonation of the PTI layers (figure 7). The stacking order for the *bulk* HCl·PTI changed from AB to AA’ (i.e. with triazine rings of adjacent layers aligned along the c-axis, with bridging amine groups alternating direction) as seen previously experimentally [16], while the protonated *surface* model structure lies between AB/AA’ stacking (electronic supplementary material, figure S8). The chloride anion position varies subtly with PTI stacking type, remaining in-plane for the protonated three-dimensional *bulk* and two-dimensional *monolayer* systems that contain a protonated site in each void (figure 6*a,c*). In the *surface* model, instead, the chlorine sits slightly below the PTI plane towards the neutral subsurface layers (figure 6*b*). Notably, there appears to be cooperative effects with protonated layers stabilized by interactions with adjacent PTI layers, whether neutral or protonated (figure 7), leading a Δ*E*_prot_ trend increasing (in modulus) with the dimensionality of the system: *bulk* (−167.9 kJ mol^−1^) < *surface* (−143.8 kJ mol^−1^) < *monolayer* (−138.9 kJ mol^−1^) < *ribbon* (−127.3 kJ mol^−1^). For real systems, where HCl is known to be capable of traversing the interlayer PTI pores [25], the implication of the cooperativity is that HCl intercalation will be encouraged throughout the entire bulk structure, instead of reacting solely on surface layers. The protonation occurring in regions away from the exposed surfaces may indeed contribute to the very slow dissolution process, observed experimentally to take place over several days (e.g. figure 3).

Protonation at NH bridge sites is less favourable than at triazine sites across all models and is coupled to the deformation from the neutral trigonal planar sp^2^ configuration of the PTI layers due to the sp^3^ hybridization of the protonated nitrogen (figure 6*d–f*). The tetrahedral nature warps the PTI layer, which is mitigated by the cooperativity effects of adjacent layers, without which (i.e. in a protonated monolayer), the warping is sufficient to make protonation endothermic (figure 7, ΔE_prot_ =  + 31.8 kJ mol^−1^).

The lower symmetry one-dimensional PTI model provides seven potential sites of protonation, allowing comparison between reactions at the pore-centric basal plane-like sites, and at positions along the edge (figure 6*g–m*). Akin to the laterally unconfined models, protonation at triazine nitrogen sites was more favourable than protonation of the hydrogenated amine/imides. The lowest energy protonation site is the triazine at the centre of the ribbon (figure 6g), most similar to triazine environments in bulk PTI, indicating a slight preference for protonation of the PTI basal plane over the edge sites, although the difference in energy to the lowest energy triazine edge site (figure 6h) is negligible (+3.3 kJ mol^−1^). Additional calculations on a three-pore-wide one dimensional *ribbon* model further reinforced this trend (electronic supplementary material, figure S9), with triazine protonation within a central pore being more favourable than edge-adjacent pore, with both more favourable than the lowest energy edge-triazine protonation. The favoured edge protonation site has the new triazine-H bond directed towards an adjacent triazine and is markedly more favourable (figure 6h, −26.0 kJ mol^−1^) than a second triazine edge site which directs its new N-H bond towards a N-H bridge along the PTI edge (figure 6*j*), highlighting the stabilization from hydrogen bonding of the added proton to an adjacent triazine (electronic supplementary material, table S3). Protonation of the terminal NH_2_ groups is less favourable than any triazine protonation (figure 6*k*, + 116.0 kJ mol^−1^ versus internal triazine protonation) but is lower in energy than reaction at either NH bridge (figure 6*l,m*), which are also associated with dramatic out-of-plane deformations of the one-dimensional ribbon (electronic supplementary material, figure S9) and endothermic Δ*E_prot_* values (electronic supplementary material, table S5).

**Figure 8.**
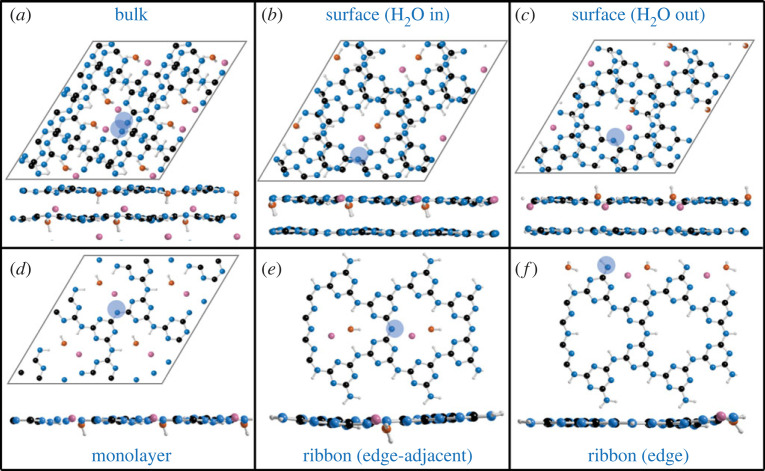
Deprotonated PTI structures from DFT calculations. (*a–d*) 2 × 2 × 1 supercell visualized down the a-axis (bottom) and c axis (top). Surface models show near-along c-axis as to align the imide bridges for clarity. Structures given for (*a*) bulk, (*b–c*) surface (*d*) monolayer, (*e–f*) 2 × 1 × 1 supercell of one-dimensional PTI ribbon model with (*e*) pore NH deprotonation and (*f*) NH2 edge terminus deprotonation. Additional surface models (directly along c-axis, and all five layers down a-axis, electronic supplementary material, figure S10) and additional ribbon models with full discussion (electronic supplementary material, figure S11) provided in electronic supplementary material.

The deprotonation of PTI with NaOH is described by the reaction (C_6_N_9_H_3_)_n_ + NaOH − > n[C_6n_N_9n_H_3n-1_^−^][Na^+^] + H_2_O, with an associated Δ*E*_deprot_ (equation (3.2)). In the basal plane, there is only a single feasible deprotonation site: the NH bridge. The resulting two-coordinate nitrogen retains the sp^2^ hybridization, leading to minimal out-of-plane distortion of the PTI sheet (figure 8). The sodium cation is located near the plane of the deprotonated PTI sheet in *bulk* system (figure 8*a*) and in the plane for less confined system (figure 8*c–f*). In all cases, the sodium cation sits near-equidistant between the deprotonated bridge's nitrogen and the nitrogen of an adjacent triazine (2.317/2.345 Å, respectively in *monolayer*, figure 8*d*, see electronic supplementary material, table S4 for all values), implying delocalization of the anionic charge across the PTI framework. The resultant water is co-intercalated with the Na (electronic supplementary material, figure S10), with the water's oxygen positioned to hydrogen bond to the two remaining N-H bridges, similar to the established H_2_O·PTI structure [24,25]. In the *surface* model, deprotonation leads to two possible states of comparable energy, dictated by the orientation of the water molecule. One O-H bond lies in the PTI's *ab* plane, allowing the other O-H to point away from the bulk (figure 8*b*, *E_deprot_* = −402.3 kJ mol^−1^), or towards the bulk (figure 8*c*, *E_deprot_* = −399.4 kJ mol^−1^). Edge deprotonation of terminal NH_2_ groups is less favourable than basal plane NH bridge deprotonation (+50.2 kJ mol^−1^), although this value also includes deintercalation of water from the PTI pore, so the true difference is likely lower and may occur along with NH bridge deprotonation in real systems (see electronic supplementary material, figure S11 for fuller discussion).

3.2 $$\Delta E_{\text{Deprot}}=\;E_{\text{NaOH}\cdot \text{PTI}}-\;E_{\text{PTI}}-\;E_{\text{NaOH}}.$$

The same cooperative effects discussed for Δ*E_prot_* can be seen in the deprotonated systems (figure 7), with stabilizing interactions between adjacent PTI layers outweighing any benefit gained from the greater flexibility of the less sterically confined sites exposed at the basal and edge surfaces. Care should be taken when directly comparing calculated Δ*E*_prot_ to Δ*E*_deprot_ values, as while Δ*E*_deprot_ is approximately 300 kJ mol^−1^ more exothermic, the solvation enthalpies of the C_6_N_9_H_2_^−^ and C_6_N_9_H_4_^+^ are not known. However, it is noted that the differences in solvation enthalpy of HCl (−74.8 kJ mol^−1^) and NaOH (−44.5 kJ mol^−1^) are significantly less than the differences between Δ*E*_prot_ and Δ*E*_deprot_. Combined with the established improved solubility of base-treated PTI over acid-treated PTI (figure 3), the notable differences between reaction energies are consistent with this trend.

## 4. Conclusion

In conclusion, carbon nitrides of poly(triazine imide) structure dissolve spontaneously at room temperature in mild acidic (HCl) and mild basic (NaOH) aqueous media as mildly negative two-dimensional species, coupled PTI basal plane pyridine-N protonation and NH-bridge deprotonation. These Brønsted reactions are preferred in the bulk over both surface layers and edge sites, due to cooperative stabilization of the charged layers from adjacent (neutral or like-charged) sheets and implies that the solvation of the monolayer sheets is highly favourable to overcome the lesser stability of charged monolayers over the (de)protonated layered crystals. The quantity of PTI in solution is highest for mildly basic solutions, with a maximum seen at pH 10.2 for LiBr·PTI and 11.4 for H_2_O·PTI, with more highly basic solutions leading to salting out of the solutions. The feasibility of water as a solvent and absence of heating/agitation leads to an intrinsically scalable, environmentally friendly route to solution processing of PTI.

## Data Availability

The datasets supporting this article have been uploaded as part of the electronic supplementary material. Supplementary material is available online [49].
